# Postmelting Encapsulation of Glass Microwires for Multipath Light Waveguiding within Phosphate Glasses

**DOI:** 10.1021/acsaom.4c00237

**Published:** 2024-07-23

**Authors:** Ioannis Konidakis, Foteini Dragosli, Aby Cheruvathoor Poulose, Josef Kašlík, Aristides Bakandritsos, Radek Zbořil, Emmanuel Stratakis

**Affiliations:** † Institute of Electronic Structure and Laser (IESL), Foundation for Research and Technology-Hellas (FORTH), 70013 Heraklion, Crete, Greece; ‡ Regional Centre of Advanced Technologies and Materials, Czech Advanced Technology and Research Institute (CATRIN), Palacký University, Šlechtitelů 27, 783 71 Olomouc, Czech Republic; § Nanotechnology Centre, Centre of Energy and Environmental Technologies, VŠB-Technical University of Ostrava, 708 00 Ostrava, Poruba, Czech Republic

**Keywords:** phosphate glass, multipath waveguides, glass microwires, silver nanoparticles, postglass melting encapsulation

## Abstract

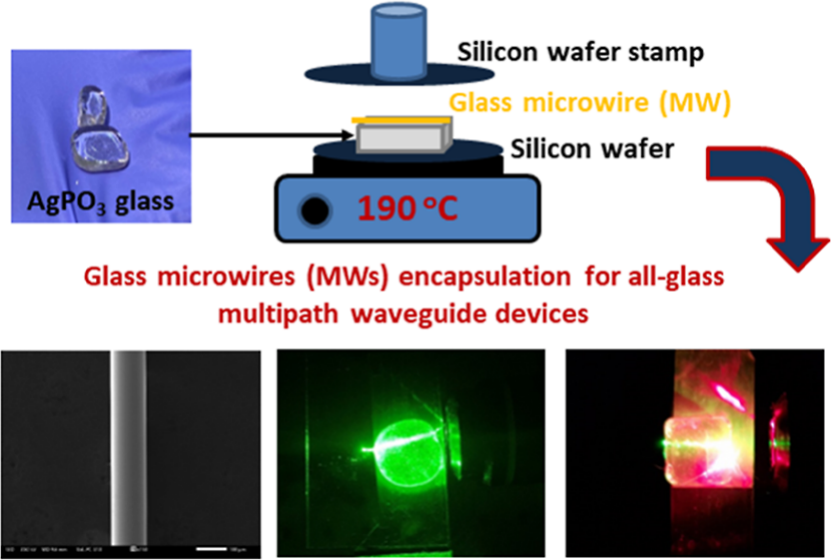

Glass waveguides are the fundamental component of advanced photonic circuits and play a pivotal role in diverse applications, including quantum information processing, light generation, imaging, data storage, and sensing platforms. Up to date, the fabrication of glass waveguides relies mainly on demanding chemical processes or on the employment of expensive ultrafast laser equipment. In this work, we demonstrate an advanced, simple, low-temperature, postmelting encapsulation procedure for the development of glass waveguides. Specifically, silver iodide phosphate glass microwires (MWs) are drawn from splat-quenched glasses. These MWs are then incorporated in a controlled manner within transparent silver phosphate glass matrices. The judicious selection of glass compositions ensures that the refractive index of the host phosphate glass is lower than that of the embedded MWs. This facilitates the propagation of light inside the encapsulated higher refractive index MWs, leading to the facile development of waveguides. Importantly, we substantially enhance the light transmission within the MWs by leveraging the plasmon resonance effects due to the presence of silver nanoparticles spontaneously generated owing to the silver iodide phosphate glass composition. Employing this innovative approach, we have successfully engineered waveguide devices incorporating either one or two MWs. Remarkably, the dual MW devices are capable of transmitting light of different colors and in multipath direction, rendering the developed waveguides outstanding candidates for extending the functionalities of diverse photonic and optoelectronic circuits, as well as in intelligent signaling applications in smart glass technologies.

## 1 Introduction

Controlling light guidance throughout transparent solids remains a continuous scientific and technological challenge in the fields of photonics and optoelectronics. In recent years many optical materials were employed in the fabrication of integrated photonic circuits and advanced waveguides, and glass remains a fundamental one among them.^1−3^ Apart from the extensive use in photonic circuits, glass waveguides expand to other numerous applications that include photodetection,^4^ light generation photonic devices,^5^ random laser and imaging,^6^ data storage components,^7,8^ quantum information processing,^9^ and sensing platforms.^10,11^

The fabrication of glass optical waveguides relies on two major approaches, a first that involves thin film deposition procedures, and a second that is based on the glass refractive index modification.^12^ The thin film deposition itself is accomplished by physical or chemical processes. Physical processes involve demanding vacuum techniques such as electron beam evaporation,^12^ and pulsed laser deposition,^13,14^ as well as sputtering methods such as magneton,^15^ and ion beam sputtering.^16^ Chemically driven thin film deposition methods could be even more demanding, as they require gas phase deposition, like vapor,^17,18^ and plasma deposition approaches,^12^ or liquid phase protocols, like sol−gel,^19,20^ and spray pyrolysis processes.^12^ Rather differently, the glass index modification is achieved by means of ion exchange,^21^ as well as, by UV and ultrafast laser patterning of the glass.^2,3,22,23^ Overall, and despite the efforts made over the years, it becomes apparent that the fabrication of glass waveguides still relies on the employment of expensive laser and optical equipment, or on demanding, and in some cases environmental unfriendly techniques for the implementation of coatings and thin films.

In recent studies we have shown the excellent feasibility of a postmelting encapsulation procedure toward the development of ultrastable and highly luminescent perovskite glasses,^24^ composite two-dimensional (2D) materials glass nanoheterojunctions,^25^ and superior photochromic glasses.^26^ Herein we exploit the postmelting protocol for the development of advanced all-glass waveguides without the need for laser processing or chemical procedures. In particular silver iodide phosphate glass microwires (MWs) are drawn from typical splat-quenched samples. Phosphate glasses are chosen due to their soft nature as expressed by their low glass transition temperature (*T*_g_), i.e. ranging below 200 °C.^24−26^ Following the drawing, the glass MWs are incorporated in a controlled manner within previously prepared transparent silver phosphate glass rectangular prisms. Notably, the MWs glass exhibits a higher refractive index than the host glass, allowing the controlled light propagation within the waveguide devices by means of total internal reflection (TIR) principles. Moreover, the proposed simple, low-temperature, postmelting procedure allows the incorporation of more than one MWs within the host glass, rendering possible the light transmission of different colors and in multiple directions. Based on this, the fabricated devices are suitable candidates for advanced photonic circuits, optoelectronic platforms, and smart sign applications.

## 2 Experimental Section

### 2.1 Development of Glasses, Glass MWs, and Waveguide Devices

**Figure 1:**
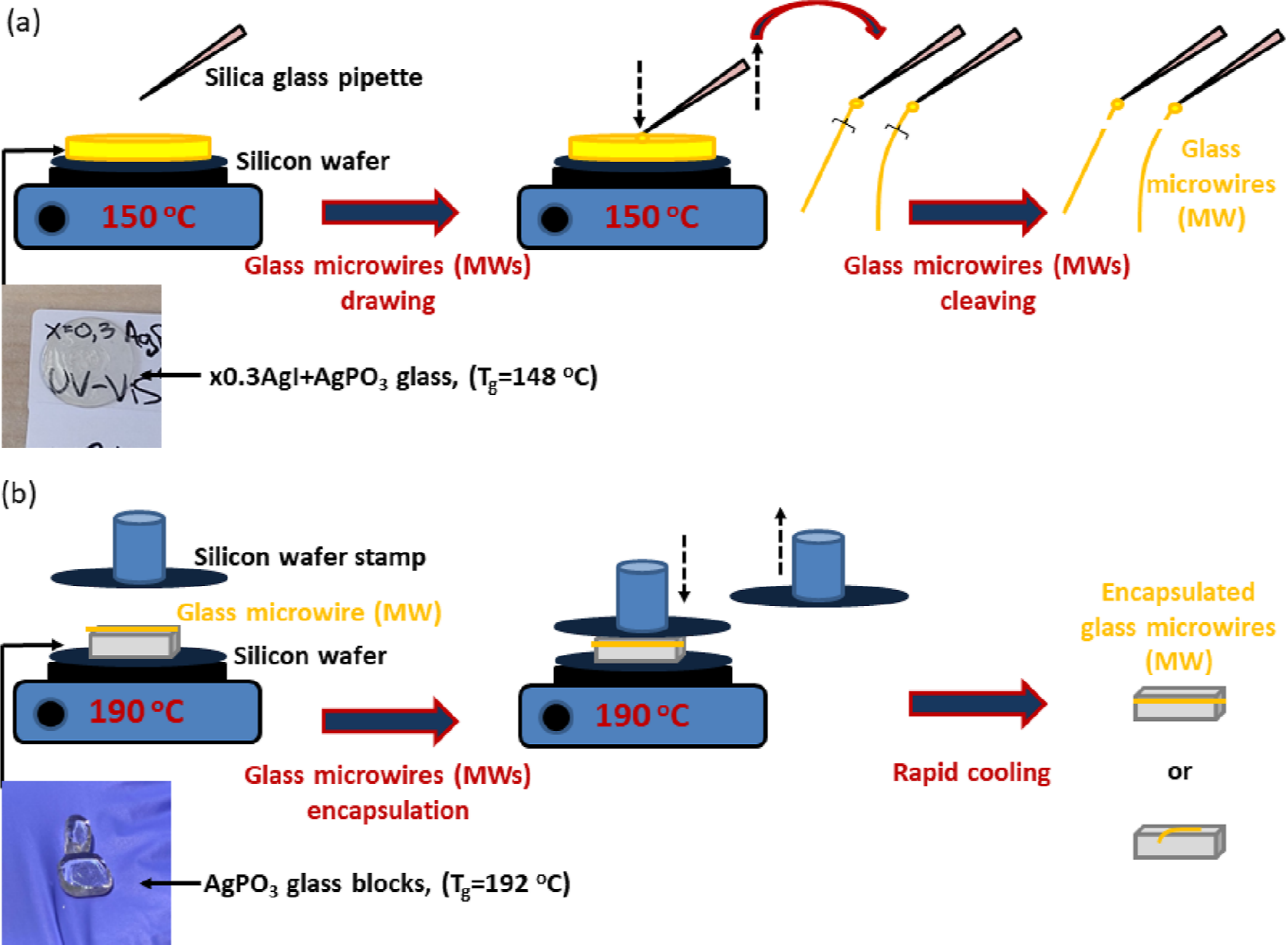
(a) Schematic representation of the glass MW drawing procedure from 0.3AgI + 0.7AgPO_3_ splat-quenched glass that is depicted in the inset photograph. (b) Schematic representation of the MWs encapsulation within the host AgPO_3_ glass. Indicative host glass prisms are depicted in the inset photograph.

For the development of the waveguides, two silver phosphate glasses within the *x*AgI + (1 −*x*)AgPO_3_ family were synthesized. Namely, the binary silver phosphate glass (AgPO_3_) with *x* = 0 was employed as the host waveguide glass, whereas the silver iodide phosphate glass with *x* = 0.3 was used for the MWs drawing. Both glasses were prepared by melting appropriate amounts of AgI, AgNO_3_, and NH_4_H_2_PO_4_ dry powders within an electrical furnace upon following a typical procedure described explicitly in our previous works.^24−27^ AgPO_3_ glasses were obtained in the form of rectangular prism blocks of various top area dimensions (0.5 × 1 cm^2^ and 1 × 1 cm^2^), after casting the melt inside custom-made molds. For improving the optical quality of the upper surface, the glasses were stamped with an optically polished silicon wafer. Figure S1 depicts indicative samples. The *x* = 0.3 glasses were splat-quenched between two silicon wafers in the form of 1 mm thick discs with a diameter of 1 cm^2^. The inset of Figure 1 presents a photograph of a typical glass disc to be used for the development of MWs.

For the MWs drawing, an as-prepared splat-quenched 0.3AgI + 0.7AgPO_3_ glass was placed on a silica wafer positioned on a heating plate. The glass was heated up to 150 °C. As the glass approaches the glass transition temperature (*T*_g_) of 148 °C,^27^ it gains viscosity. Immediately after, a tip of a typical glass pipet was emersed within the sample and moved upward as depicted schematically in Figure 1a. The melted glass sticks to the tip of the pipet, and as the pipet moves away from the splat-quenched glass a MW was drawn as demonstrated in Video S1. The MW is then removed from the end face of the pipet, whereas the speed of removing the pipet from the glass determines the MW thickness.

After drawing, the 0.3AgI + 0.7AgPO_3_ glass MWs were cleaved to the appropriate length in order to be encapsulated within the host binary AgPO_3_ glass. As shown in Figure 1b the host glass is positioned on a silica wafer placed on a heating plate. The inset of Figure 1b depicts photos of typical AgPO_3_ glass rectangular prisms that are employed for the fabrication of the waveguide devices. On the upper surface of the host glass, the MW is placed at the intended immersing position as shown in Figure 1b. The temperature rises up to 190 °C, i.e. close to the *T*_g_ of the binary glass (192 °C).^24^ Once the host glass gains viscosity the MWs is pressed from the top so that is totally immersed within the host glass (Figure 1b). Immediately after, the silicon wafer is removed from the heating plate, and the waveguide composite glass device cools down to room temperature. Notably, the same route applies for single and multipath waveguide devices were more than one MWs are incorporated at different directions within the same host phosphate glass prism.

### 2.2 Materials Characterization

A scanning electron microscope (JEOL, JSM-7000F) was employed for the morphological examination of the synthesized glasses and the waveguide devices, along with scanning transmission electron microscopy (STEM) studies in the high-angle annular dark field (HAADF) mode. The elemental mapping was performed with an FEI TITAN G2 60-300 HRTEM microscope with an X-FEG type emission gun, operating at 300 kV, objective-lens image spherical aberration corrector and ChemiSTEM EDS detector. For the optical properties, ultraviolet–visible (UV–vis) absorption spectra were collected on a Cary 50 UV–vis spectrophotometer (Varian). The structural modifications of the synthesized glasses were studied by means of Raman spectroscopy. Namely, room temperature Raman spectra with a resolution of 1 cm^−1^ were collected at the backscattering geometry upon employing a 532 nm laser line for excitation.^24,25^

The waveguiding features of the developed single MW glass waveguides were demonstrated upon employing three distinct light emission sources. In particular, a blue cw laser emitting at 450 nm, a green cw laser emitting at 526 nm, and a red cw laser emitting at 680 nm were used. Figure S2a presents the experimental set up with the necessary optical components to guide the selected beam toward the waveguide device at one at a time manner. A microscope objective mounted on an *x*, *y*, *z* stage was employed to assist the coupling of light within the encapsulated MW of the waveguide (Figure S2a). For the multipath waveguide devices, the green and red laser were used. The green laser is coupled within the parallel to the edges of the glass MW as shown for the single MW devices in Figure S2a. In addition, a convex lens from the opposite direction is employed to focus the red laser beam on the second MW (Figure S2b), which is incorporated parallel or diagonally to the other MW that waveguides the green light throughout the glass. A typical power meter was used to determine the transmission losses of the fabricated waveguides, upon comparing the power of the incident light with that of the output light on the other side of the waveguides (Figure S2a).

## 3 Results and Discussion

**Figure 2:**
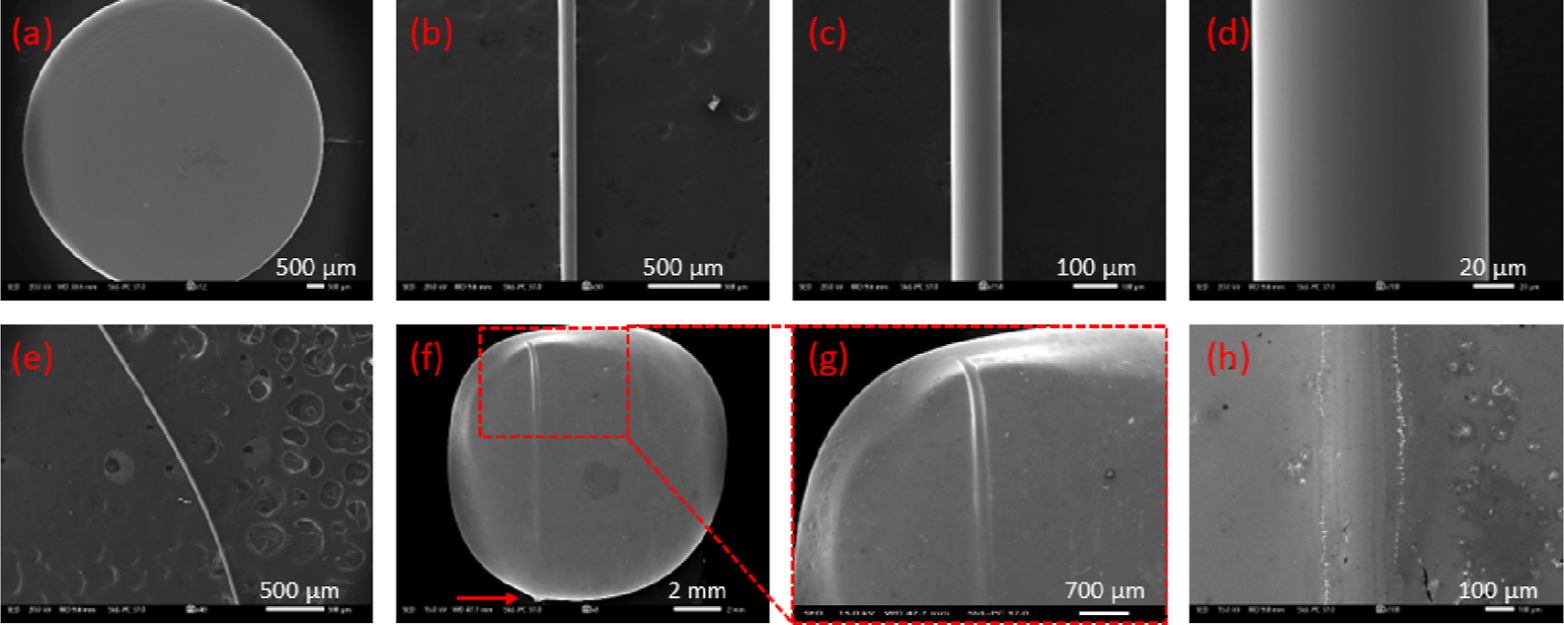
(a) SEM images of the 0.3AgI + 0.7AgPO_3_ splat-quenched glass. (b) Typical SEM image of a glass MW drawn from the splat-quenched glass. (c,d) Higher magnification images of the same glass MW. (e) SEM image of a curved MW. (f) SEM image of a single MW waveguide device upon encapsulation of the MW within the host glass. (g) Magnified area of the device. (h) Magnified area of the MW immersion point on the surface of the host glass.

Figure 2a presents a scanning electron microscopy (SEM) image of the splat-quenched 0.3AgI + 0.7AgPO_3_ glass that was used for drawing the MWs. Figure 2b–d depict typical SEM top-view photos of a drawn MW with a diameter of 100 μm. It becomes apparent that the employed method results to the formation of uniform glass MWs with smooth and homogeneous surface, while their diameter could range between 20 and 200 μm. The same applies to thinner curved MWs (Figure 2e), that were drawn upon following the same procedure. Figure 2f shows the upper surface of the AgPO_3_ host glass upon encapsulation of the 100 μm glass MW, whereas Figure 2g presents a magnified area. Medium size diameter MWs were selected for the waveguide fabrication, since that simplifies both the postmelting encapsulation and the light coupling. Inspection of Figure 2g reveals that the MW is immersed within the host glass at a depth of around 200 μm, while leaving a notable mark on the surface of the host glass block. A typical SEM micrograph of the immersion point across the surface of the host glass is depicted in Figure 2h. Notably, following the incorporation of the glass MW, the surface of the host glass remains smooth. As it is depicted in Figure 2f, at the upper side of the waveguide device the incorporated glass MW has been cleaved exactly on the end face of the glass to facilitate light coupling. Rather differently, at the output side of the device, as pointed out by the red arrow in Figure 2f, the encapsulated glass MW was cleaved at an exceeding length of around 80 μm, in order to demonstrate better and monitor the light transmission through it, when the waveguiding features of the devices are studied.

It is noted that following the developed postmelting procedure the encapsulation of the higher refractive index glass MW inside the host glass is realized while the MW remains intact, despite its lower *T*_g_ when compared to that of the host glass. The reason behind this lies on the fact that the cylindrical MW is originally placed on the surface of the host glass block (Figure 1b). The latter glass is heated near to its *T*_g_ to gain viscosity and facilitate the immersion of the MW. However, due to the shape and size of the MW, the contact area to the host glass is minimal and thus the heat transfer to the MW is inefficient to exceed the 148 °C that would make the MW melt. Moreover, the MW is ventilated from the top. This was the case for all fabricated samples upon visual inspection during the encapsulation procedure, while evidence of this appears also on the SEM image of Figure 2f, that depicts the end face of the incorporated 0.3AgI + 0.7AgPO_3_ glass MW remaining intact and exceeding the edge of the binary AgPO_3_ host glass. However, in the interior of the host glass the softer glass MW would melt, creating a horizontal pathway of silver-rich higher refractive index glass within the lower refractive index host glass, as depicted schematically in Figure S3.

**Figure 3:**
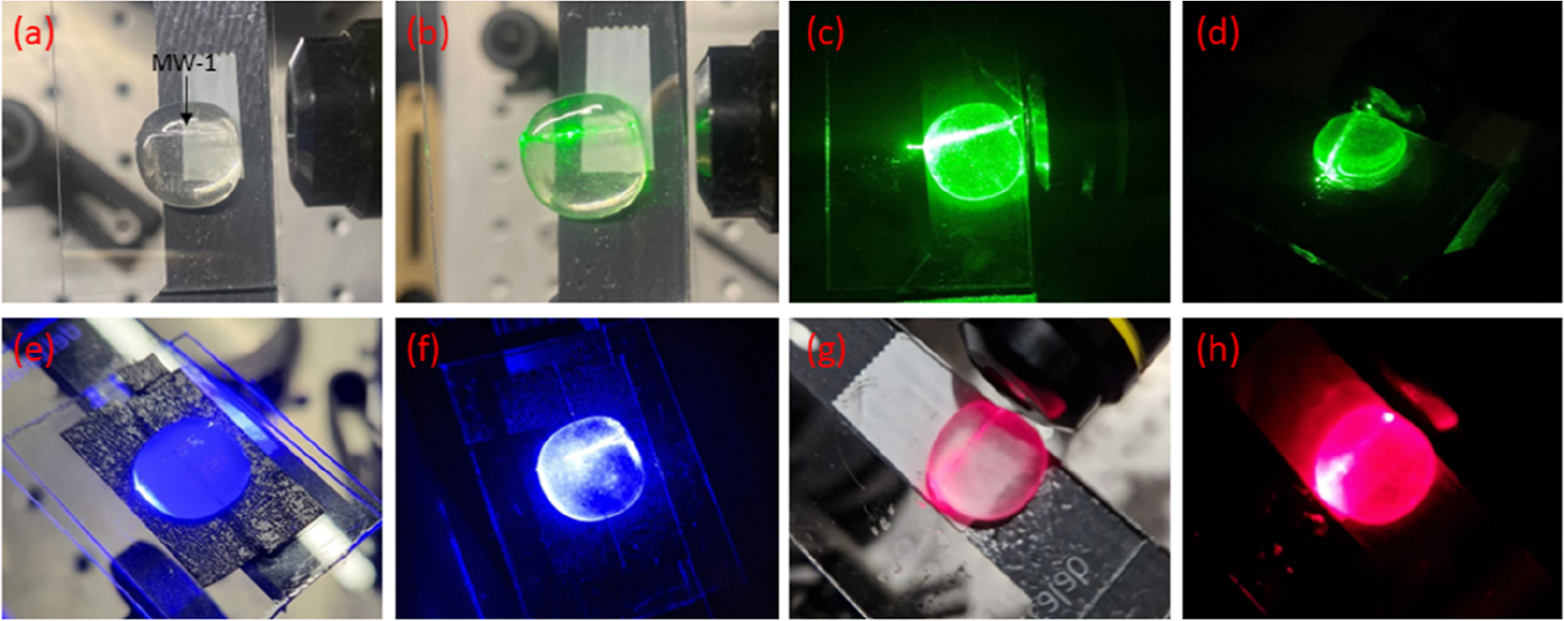
(a) Optical microscope photo of a typical single MW waveguide device, in which the MW immersion point is visible. Waveguiding features of the green laser with lights-on (b), and under dark (c). (d) Picture of the same waveguide device under different angle, so that the output point is more visible. Waveguiding features of the blue laser with lights-on (e), and under dark (f). Waveguiding features of the red laser with lights-on (g), and under dark (h).

The outcome of forming the silver-rich pathway along the AgPO_3_ glass in terms of waveguiding becomes immediately apparent in Figure 3. Figure 3a shows an optical microscopy photo of the studied waveguide device, whereas Figure 3b–h present the waveguide features of the green (Figure 3b–d), blue (Figure 3e,f), and red (Figure 3g,h) light upon coupling in the beams of the corresponding cw lasers. The employed input power of the three cw laser sources is 1.9 mW. The first photo of each color depicts the devices with lab lights on, whereas the other show the devices under dark conditions. Upon achieving a suitable incident light angle to couple light in the higher refractive index silver-rich channel the light propagates through the host glass, toward the other side of the device. Indicatively, inspection of Figure 3d,f reveal bright spots on the end-face tip that was left to exceed the exit of the waveguide device (Figure 2f).

In order to couple light in the waveguide efficiently, the focused incident beam was moved through an objective mounted on a *x*, *y*, *z* micrometric stage (Figure S2), while the glass was slightly tilted in front of the objective, so that an incident light angle is formed. To demonstrate this, Figure S4a presents an example of the red laser beam focused outside the waveguide pathway. The red and blue dotted lines in the same figure, point out that some of the light continues uncontrollably to the other side of the glass, i.e. without waveguiding features. On the contrary, once the beam is moved via the micrometric stage within the waveguide core, it waveguides through it, and deviates from the vertical orientation with respect to the objective (Figure S4b). Similarly, Video S2 illustrates the coupling-in light process for the green laser. At the start of the video (first 2 s), the green laser beam is focused outside the waveguide pathway, and no light transmits through the glass to the other side of the waveguide. As the video progresses, the beam is moved toward the waveguide channel. Once the beam reaches the entrance point at 3 s onward, the light propagation occurs, as it can be seen from the luminescence at the other side of the waveguide device. In both red and green light demonstrations, the glass is positioned exactly at the same place throughout the process, i.e. stacked on a microscope slide with double sided tape (Figure S4).

The required incident light angle for efficient light transmission through the fabricated waveguides is rationalized in terms of the TIR principles.^28,29^ In general, TIR is the phenomenon in which light that is traveling from a denser medium to a rarer medium is reflected in the former medium at the interface of the two media (Figure S3). However, there are two necessary conditions for the occurrence of TIR between two glass optical media, as is the case in optical fiber architectures. First, the light must be traveling from a denser medium to a sparser medium, i.e. the refractive index of the former glass must be higher than that of the latter. Second, the light angle of incident must be greater than the critical angle that is determined from Snell’s law.^28,29^ Notably, both conditions are valid in the case of the studied waveguides. In particular, the refractive index of the denser silver-rich channel would be of 1.90,^27^ whereas the corresponding value for the host glass that surrounds the waveguiding pathway is of 1.79 (Figure S3).^27^ Moreover, the critical angle for TIR in the so-formed waveguides is determined equal to 70.4°. As depicted in Figure 3, the employed incident light angles are greater than the critical angle of 70.4°. Furthermore, to provide a reference in terms of the optical losses of the fabricated waveguide devices, we have studied extensively the output power of light with respect to the power of coupled in light upon employing the cw green laser. Figure S5 presents the determined transmission losses of a typical single MW device for various applied powers. The average loss was determined equal to 3.2 db/cm, whereas the minimum obtained loss of 1.9 db/cm at the maximum employed power of 1.9 mW was considerably low.

**Figure 4:**
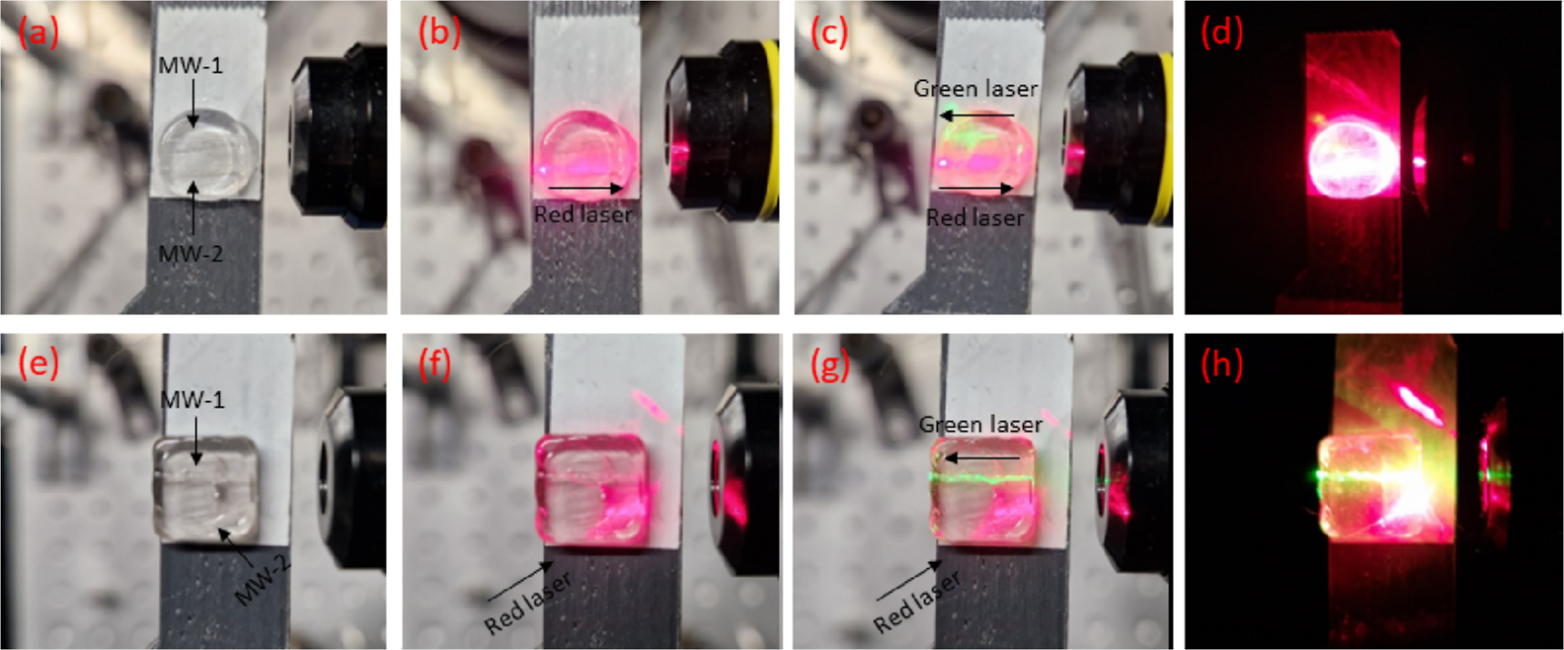
(a) Optical microscope photo of a multipath waveguide, in which two parallel MWs are incorporated. The arrows point out the two immersion points. (b) Red laser waveguiding throughout one of the incorporated MWs (left to right direction). (c) Red and green (right to left) laser waveguiding throughout the two parallel MWs. (d) Same as previous under dark. (e) Optical microscope photo of a multipath waveguide, in which the second MW is encapsulated diagonally with respect to the other. (f) Red laser transmission through the diagonally placed MW (bottom to right direction). (g) Red and green (right to left) light transmission through the diagonally and parallel placed MWs. (h) Same as previous under dark.

Along similar lines, Figure 4 presents the operation of the multipath waveguides upon simultaneously transmitting green and red light. Figure 4a–d depict a device in which two MWs are incorporated parallel to each other, whereas Figure 4e–h show a waveguide glass in which the second MW is encapsulated in a diagonal direction, i.e. facing on the lower hand side of the host glass square rather than the opposite end face. Inspection of the optical microscope photos of the devices shown in Figure 4a,e reveal the obvious marks of the two immersed MWs within the host glasses. The corresponding SEM photos of the multipath waveguide devices are shown in Figure S6a,b. Figure 4b presents the single red-light transmission within one MW, whereas as Figure 4c,d depict the corresponding photos in which both red and green light are waveguided within the parallel pathways of the device, upon coupling light in from the opposite directions (Figure S2). On the same manner, Figure 4f depicts the single transmission of red light through the diagonally formed pathway, and Figure 4g,h show the corresponding photos in which both green and red light are waveguided through the glass. Finally, Figure S6c,d depict photos in which only the green laser is coupled in the devices. Overall, the captured photos demonstrate explicitly the two distinct waveguiding pathways formed following the encapsulation of the two MWs within the host glass.

**Figure 5:**
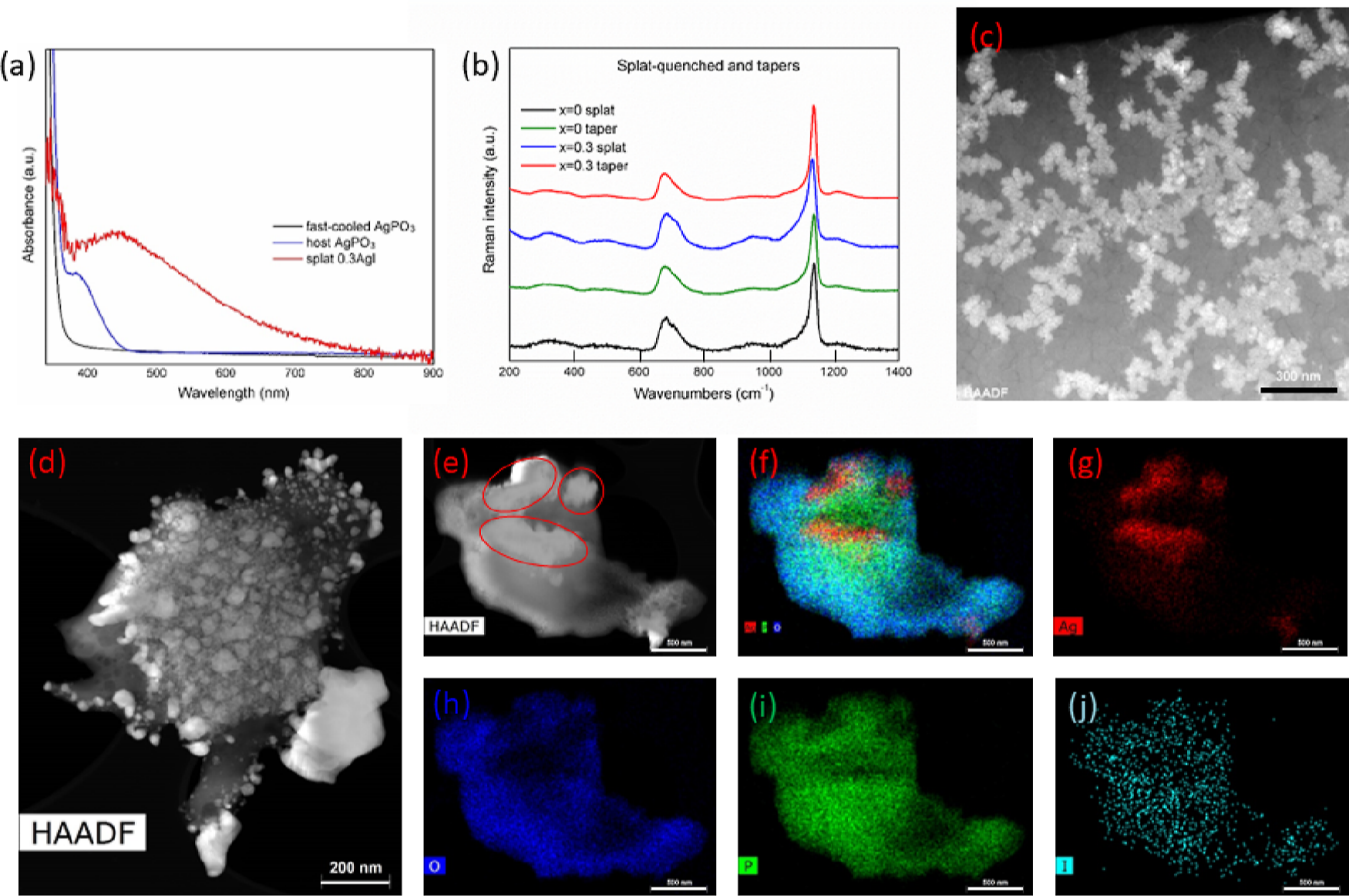
Optical absorbance (a), and Raman (b) of the employed glasses. STEM photo of the silver nanoparticles (AgNPs) within the host AgPO_3_ glass (c), and the 0.3AgI + 0.7AgPO_3_ MWs glass (d). (e) HAADF image of the agglomerated AgNPs within the MWs glass. (f) HRTEM-EDX elemental mapping of the same sample, along with the individual element spatial analysis for Ag (g), O (h), P (i), and I (j).

The optical and structural properties of the employed glasses were also assessed. Figure 5a presents the optical absorbance of the AgPO_3_ host glass and the splat-quenched 0.3AgI + 0.7AgPO_3_ glass that was used for drawing the MWs. For the sake of comparison, a corresponding spectrum of a fast-cooled AgPO_3_ splat-quenched glass is also shown. Inspection of Figure 5a reveals characteristic absorbance peaks at the 400 to 600 nm range for the host and the MW glasses, attributed to the presence of silver nanoparticles (AgNPs). Notably, the profile of the latter glass is more intense and shifted to longer wavelengths indicative for the formation of more and with wider size distribution AgNPs, upon the introduction of AgI.^30^ On the contrary, such profile is absent from the corresponding profile of the ultrafast cooled glass, in which the formation of AgNPs is suppressed due to the rapid cooling.

The effect of the AgNPs presence on the structure of the employed phosphate glasses was investigated by Raman spectroscopy. Figure 5b depicts the normalized Raman spectra of both AgPO_3_ and 0.3AgI + 0.7AgPO_3_ glasses in the forms of splat-quenched discs and drawn MWs. It is known that the metaphosphate network consists mainly of chains upon connecting phosphate tetrahedral units with bridging and nonbridging (terminal) oxygen atoms.^24,26^ The obtained two main Raman features of Figure 5b originate from these structural units. Indeed, the key band at 1140 cm^−1^ is attributed to the symmetric stretching vibration of the terminal PO_2_^−^ groups, whereas the broader band at 675 cm^−1^ arises from the symmetric stretching of the P–O–P bridges of the phosphate backbone.^24,26^ The relative intensities of the two bands provides direct evidence on the population of the phosphate entities, and thus, on the phosphate network modifications. However, the analysis of Figure 5b reveals no significant changes in the relative intensities of the Raman features between splat-quenched glasses and drawn MWs. Thus, it is concluded that despite the MW drawing process and the random formation of AgNPs upon the introduction of AgI, the phosphate network is practically intact, rendering the employed glasses suitable for the fabrication of the studied waveguide devices.

STEM offers useful information on the morphology of the so-formed AgNPs within the host glass and the embedded MWs. Figure 5c presents an indicative STEM photo of the AgNPs within the host glass, whereas Figure 5d shows the corresponding image of AgNPs within the MWs glass. Inspection of the two samples reveals that the addition of AgI in the latter glass causes the agglomeration of AgNPs and the formation of larger size nanoclusters and particles. Data analysis of the AgNPs size distribution within the host AgPO_3_ glass (Figure 5c) indicates an average size of 20 nm. Rather differently, once the AgI is introduced for the MWs glass (Figure 5d), the average AgNPs size increase up to 40 nm, while silver agglomeration results to for the formation of clusters up to 500 nm (Figure 5e). Figure 5f–j depict the elemental mapping of the sample by means of HRTEM, corresponding to the combined and to the individual maps for Ag, O, P, and I. The highlighted cluster regions in Figure 5e, exhibit dominant Ag presence, whereas in the same regions significant absence of the other elements is noted. The presence of AgNPs within the silver-rich waveguide pathway of the fabricated devices, accounts plausibly for the obtained low losses (Figure S5). It is well-known that AgNPs absorb and scatter light with great efficiency. Indeed, they are often used as active centers for improving light absorption and transmission in various types of devices, that include photovoltaics,^31^ and fibers for random lasers and light emitting diodes.^32^ In particular, AgNPs interact strongly with light since the conduction electrons on the metal surface exhibit a collective oscillation after light excitation, known as localized surface plasmon resonance (LSPR). The existence of LSPR along with the matching of emission in the visible spectra range, amplifies the waveguided emission of light, and minimizes optical loss throughout the waveguide.^32^ Apart from this, the presence of smaller and less AgNPs within the host glass block, accounts plausibly for the obtained random scattering points that can be seen in Figure 3b,f, outside the silver-rich pathway of the waveguide.

## 4 Conclusions

We have shown the fabrication of advanced waveguides upon incorporating silver-rich glass MWs within a phosphate glass host, by means of a simple, fast, postmelting encapsulation procedure. The glass MWs were drawn from typical splat-quenched glasses, while exhibiting higher refractive index than the host glass. Upon embedding them within the host glass, a pathway of higher refractive index is formed, creating suitable conditions for TIR across the waveguide. Moreover, the developed protocol allows the incorporation of multiple MWs within the same host, allowing simultaneously guiding features of different light sources and at different directions. Overall, we believe that the proposed method poses important technological advancements and opens the doors for the industrial scale production of glass waveguides and composites glasses for various photonic circuits, optoelectronic, and smart sign applications. This is feasible upon employing widely used large scale industrial glass melting procedures and post melting treatment techniques, and MW drawing facilities equivalent to these of optical fiber making.

## Supporting Information Available

Photographs and SEM images of so-formed glasses and waveguides, schematics of the experimental set up and coupling mechanism, optical losses analysis (PDF)
Glass MW drawing process (MP4)
Demonstration of the light coupling-in process and waveguide operation (MP4)

## References

[ref1] El Hassan A.; Kunst F. K.; Moritz A.; Andler G.; Bergholtz E. J.; Bourennane M. Corner States of Light in Photonic Waveguides. Nat. Photonics 2019, 13, 697–700. 10.1038/s41566-019-0519-y.

[ref2] Tan D.; Wang Z.; Xu B.; Qiu J. Photonic Circuits Written by Femtosecond Laser in Glass: Improved Fabrication and Recent Progress in Photonic Devices. Adv. Photonics 2021, 3, 024002 10.1117/1.AP.3.2.024002.

[ref3] Tan D.; Zhang B.; Qiu J. Ultrafast Laser Direct Writing in Glass: Thermal Accumulation Engineering and Applications. Laser Photonics Rev. 2021, 15, 2000455 10.1002/lpor.202000455.

[ref4] Goldstein J.; Lin H.; Deck-off-Jones S.; Hempel M.; Lu A.-Y.; Richardson K. A.; Palacios T.; Kong J.; Hu J.; Englund D. Waveguide-Integrated Mid-Infrared Photodetection Using Graphene on a Scalable Chalcogenide Glass Platform. Nat. Commun. 2022, 13, 3915 10.1038/s41467-022-31607-7. 35798746 PMC9262905

[ref5] Zhong L.; Wang Y.; Tan D.; Qiu J. Toward 3D Integration of Highly See-Through Photonic Circuits in Glass. Laser Photonics Rev. 2023, 17, 2200767 10.1002/lpor.202200767.

[ref6] Dey A.; Pramanik A.; Biswas S.; Chatterjee U.; Kumbhakar P. Tunable and Low-Threshold Random Lasing Emission in Waveguide Aided Rhodamine-6G Dye Incorporated Silica Embedded Thin Films. J. Lumin. 2022, 251, 119252 10.1016/j.jlumin.2022.119252.

[ref7] Liu Y.; Zhu B.; Dai Y.; Qiao X.; Ye S.; Teng Y.; Guo Q.; Ma H.; Fan X.; Qiu J. Femtosecond Laser Writing of Er^3+^-Doped CaF_2_ Crystalline Patterns in Glass. Opt. Lett. 2009, 34, 3433–3435. 10.1364/OL.34.003433. 19881618

[ref8] Pitwon R.; Wang K.; Yamauchi A.; Ishigure T.; Schröder H.; Neitz M.; Singh M. Competitive Evaluation of Planar Embedded Glass and Polymer Waveguides in Data Center Environments. Appl. Sci. 2017, 7, 940 10.3390/app7090940.

[ref9] Stone A.; Jain H.; Dierolf V.; Sakakura M.; Shimotsuma Y.; Miura K.; Hirao K.; Lapointe J.; Kashyap R. Direct Laser-Writing of Ferroelectric Single-Crystal Waveguide Architectures in Glass for 3D Integrated Optics. Sci. Rep. 2015, 5, 10391 10.1038/srep10391. 25988599 PMC4437375

[ref10] Xiao-hong Z.; Lan-hua L.; Wei-qi X.; Bao-dong S.; Jian-wu S.; Miao H.; Han-chang S. A Reusable Evanescent Wave Immunosensor for Highly Sensitive Detection of Bisphenol A in Water Samples. Sci. Rep. 2014, 4, 4572 10.1038/srep04572. 24699239 PMC3975238

[ref11] Liu L.; Zhou X.; Wilkinson J. S.; Hua P.; Song B.; Shi H. Integrated Optical Waveguide-Based Fluorescent Immunosensor for Fast and Sensitive Detection of Microcystin-LR in Lakes: Optimization and Analysis. Sci. Rep. 2017, 7, 3655 10.1038/s41598-017-03939-8. 28623299 PMC5473886

[ref12] Righini G.; Chiappini A. Glass Optical Waveguides: A Review of Fabrication Techniques. Opt. Eng. 2014, 53, 071819 10.1117/1.OE.53.7.071819.

[ref13] Martino M.; Caricato A. P.; Fernandez M.; Leggieri G.; Jha A.; Ferrari M.; Mattarelli M. Pulsed Laser Deposition of Active Waveguides. Thin Solid Films 2003, 433, 39–44. 10.1016/S0040-6090(03)00310-9.

[ref14] Irannejad M.; Pasha M.; Jose G.; Steenson P.; Fernandez T. T.; Jha A.; Jiang Q.; Zhang Z. Y.; Hogg R. A.; Evans C.; et al. Active Glass Waveguide Amplifier on GaAs by UV-Pulsed Laser Deposition and Femtosecond Laser Inscription. Laser Phys. Lett. 2012, 9, 329–339. 10.7452/lapl.201110101.

[ref15] Anne M.-L.; Keirsse J.; Nazabal V.; Hyodo K.; Inoue S.; Boussard-Pledel C.; Lhermite H.; Charrier J.; Yanakata K.; Loreal O.; Le Person J.; Colas F.; Compere C.; Bureau B. Chalcogenide Glass Optical Waveguides for Infrared Biosensing. Sensors 2009, 9, 7398–7411. 10.3390/s90907398. 22423209 PMC3290500

[ref16] de Sande J. C. G.; Afonso C. N.; Escudero J. L.; Serna R.; Catalina F.; Bernabeu E. Optical Properties of Laser-Deposited a-Ge Films: A Comparison with Sputtered and e-Beam Deposited Films. Appl. Opt. 1992, 31, 6133–6138. 10.1364/AO.31.006133. 20733819

[ref17] Saito T.; Hanada T.; Kitamura N.; Kitamura M. Photosensitivity in Silica-Based Waveguides Deposited by Atmospheric Pressure Chemical Vapor Deposition. Appl. Opt. 1998, 37, 2242–2244. 10.1364/AO.37.002242. 18273148

[ref18] Ay F.; Aydinli A.; Agan S. Low-Loss as-Grown Germanosilicate Layers for Optical Waveguides. Appl. Phys. Lett. 2003, 83, 4743–4745. 10.1063/1.1631753.

[ref19] Goncalves R. R.; Carturan G.; Zampedri L.; Ferrari M.; Montagna M.; Chiasera A.; Righini G. C.; Pelli S.; Ribeiro S. J. L.; Messaddeq Y. Sol-Gel Er-Doped SiO_2_-HfO_2_ Planar Waveguides: A Viable System for 1.5 μm Application. Appl. Phys. Lett. 2002, 81, 28–30. 10.1063/1.1489477.

[ref20] Peled A.; Chiasera A.; Nathan M.; Ferrari M.; Ruschin S. Monolithic Rare-Earth Doped Sol-Gel Tapered Rib Waveguide Laser. Appl. Phys. Lett. 2008, 92, 221104 10.1063/1.2936961.

[ref21] Righini G. C.; Linares J. Active and Quantum Integrated Photonic Elements by Ion Exchange in Glass. Appl. Sci. 2021, 11, 5222 10.3390/app11115222.

[ref22] Ams M.; Marshall G. D.; Dekker P.; Piper J. A.; Withford M. J. Ultrafast Laser Written Active Devices. Laser Photonics Rev. 2009, 3, 535–544. 10.1002/lpor.200810050.

[ref23] Meany T.; Grafe M.; Heilmann R.; Perez-Leija A.; Gross S.; Steel M. J.; Withford M. J.; Szameit A. Laser Written Circuits for Quantum Photonics. Laser Photonics Rev. 2015, 9, 363–384. 10.1002/lpor.201500061.

[ref24] Konidakis I.; Brintakis K.; Kostopoulou A.; Demeridou I.; Kavatzikidou P.; Stratakis E. Highly Luminescent and Ultrastable Cesium Lead Bromide Perovskite Patterns Generated in Phosphate Glass Matrices. Nanoscale 2020, 12, 13697–13707. 10.1039/D0NR03254A. 32573581

[ref25] Sarkar A. S.; Konidakis I.; Demeridou I.; Serpetzoglou E.; Kioseoglou G.; Stratakis E. Robust B-Exciton Emission at Room Temperature in Few-Layers of MoS_2_:Ag Nanoheterojunctions Embedded into a Glass Matrix. Sci. Rep. 2020, 10, 15697 10.1038/s41598-020-72899-3. 32973224 PMC7518262

[ref26] Adamidis M.; Konidakis I.; Stratakis E. Post-Glass Melting Synthesis and Photochromic Properties of AgCl-AgPO_3_ glasses. J. Mater. 2023, 9, 455–463. 10.1016/j.jmat.2022.12.006.

[ref27] Konidakis I.; Pissadakis S. Optical Spectra Tuning of All-Glass Photonic Bandgap Fiber Infiltrated with Silver Fast-Ion-Conducting Glasses. Materials 2014, 7, 5735–5745. 10.3390/ma7085735. 28788157 PMC5456184

[ref28] Barnoski M. K. Fundamentals of Optical Fiber Communications; Elsevier, 1981.

[ref29] Rioux M.; Ledemi Y.; Morency S.; de Lima Filho E. S.; Messaddeq Y. Optical and Electrical Characterizations of Multifunctional Silver Phosphate Glass and Polymer-Based Optical Fibers. Sci. Rep. 2017, 7, 43917 10.1038/srep43917. 28256608 PMC5335562

[ref30] Zong R.; Wang X.; Shi S.; Zhu Y. Kinetically Controlled Seed-Mediated Growth of Narrow Dispersed Silver Nanoparticles up to 120 nm: Secondary Nucleation, Size Focusing, and Ostwald Ripening. Phys. Chem. Chem. Phys. 2014, 16, 4236–4241. 10.1039/c3cp54846e. 24452515

[ref31] Nourolahi H.; Behjat A.; Hosseini-Zarch S. M. M.; Bolorizadeh M. A. Silver Nanoparticle Plasmonic Effects on Hole-Transport Material-Free Mesoporous Heterojunction Perovskite Solar Cells. Sol. Energy 2016, 139, 475–483. 10.1016/j.solener.2016.10.023.

[ref32] Chen W.-C.; Shiao J.-H.; Tsai T.-L.; Jiang D.-H.; Chen L.-C.; Chang C.-H.; Lin B.-H.; Lin J.-H.; Kuo C.-C. Multiple Scattering from Electrospun Nanofibers with Embedded Silver Nanoparticles of Tunable Shape for Random Lasers and White-Light-Emitting Diodes. ACS Appl. Mater. Interfaces 2020, 12, 2783–2792. 10.1021/acsami.9b16059. 31869205

