# Supplementary material for: Postmelting Encapsulation of Glass Microwires for Multipath Light Waveguiding within Phosphate Glasses
Source: ACS Appl Opt Mater. 2024 Jul 23;2(8):1636–43. doi: 10.1021/acsaom.4c00237 (PMC11348410; doi:10.1021/acsaom.4c00237)
Supplement: Supplementary file 1 — ot4c00237_si_001.pdf [file ot4c00237_si_001.pdf]

Supporting Information

Post-melting encapsulation of glass microwires for multipath light waveguiding within phosphate glasses

Ioannis Konidakis*[1], Foteini Dragosli[1], Aby Cheruvathoor Poulose[2], Josef Kašlík[2], Aristides Bakandritsos[2,3], Radek Zbořil[2,3] and Emmanuel Stratakis*[1]

1. Institute of Electronic Structure and Laser (IESL), Foundation for Research and Technology-Hellas (FORTH), 70013 Heraklion-Crete, Greece.
2. Regional Centre of Advanced Technologies and Materials, Czech Advanced Technology and Research Institute (CATRIN), Palacký University, Šlechtitelů 27, 783 71, Olomouc, Czech Republic.
3. Nanotechnology Centre, Centre of Energy and Environmental Technologies, VŠB-Technical University of Ostrava, Ostrava-Poruba, Czech Republic.

***Corresponding authors:** ikonid@iesl.forth.gr, stratak@iesl.forth.gr

Figures:

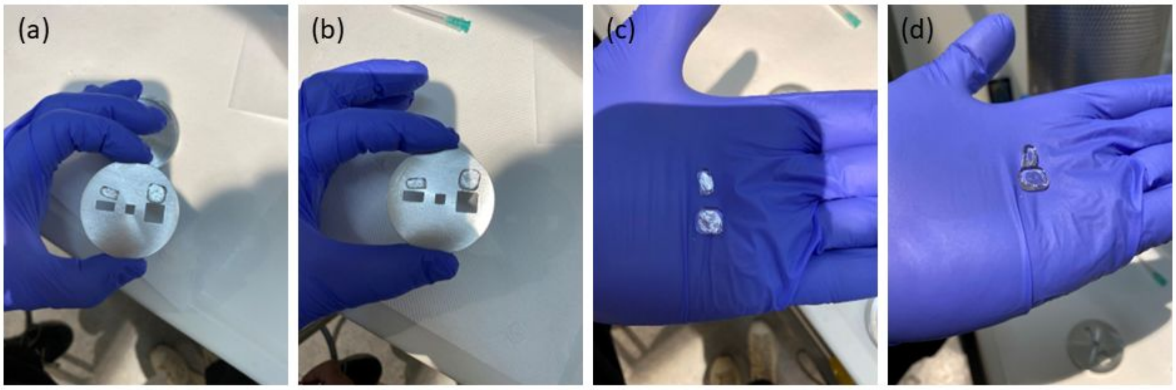

Fig. S1: Typical host $AgPO_3$ glasses obtained in the form of rectangular prism blocks of various top area dimensions (0.5 x 1 cm^2 and 1 x 1 cm^2), after casting the melt inside custom made moulds.

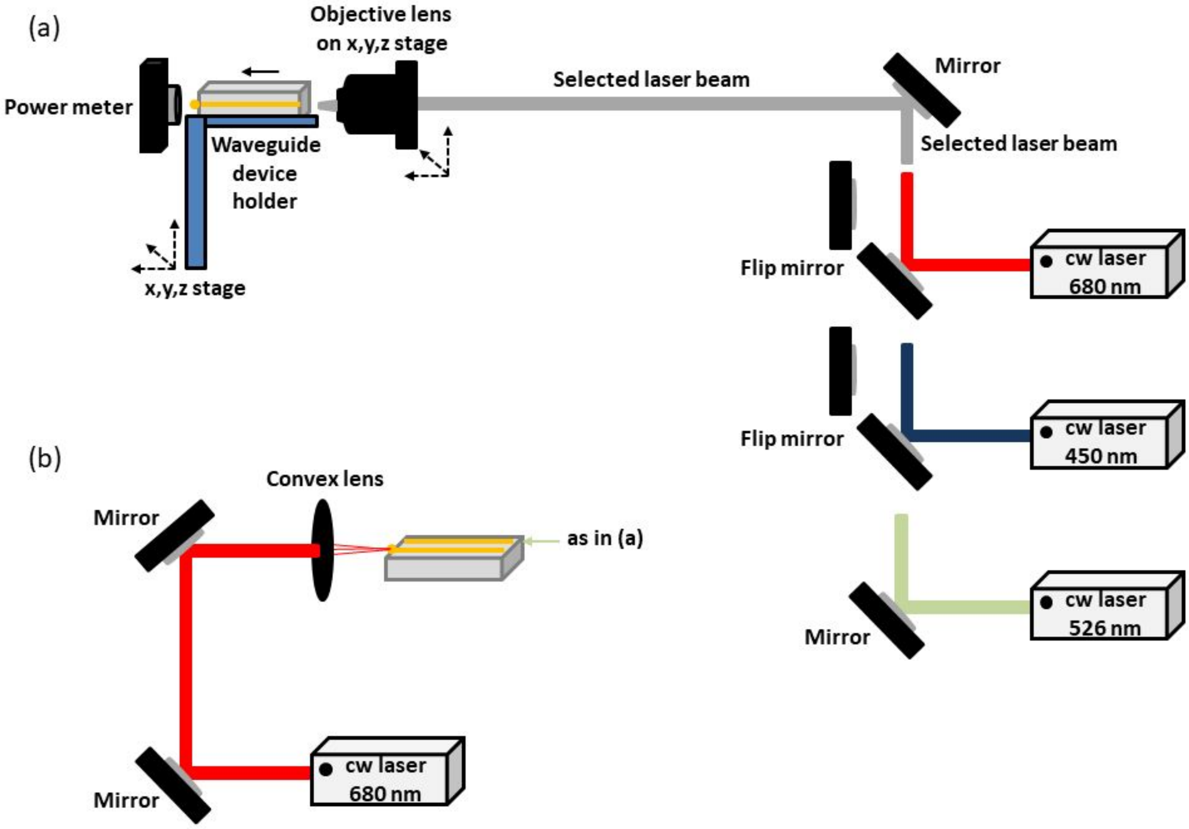

Fig. S2: (a) Depiction of experimental set up with the necessary optical components for guiding the selected beam towards the waveguide device at one at a manner. **(b)** Convex lens configuration for focusing the red laser beam on the second microwire (MW), i.e. incorporated either parallelly or diagonally to other MWs.

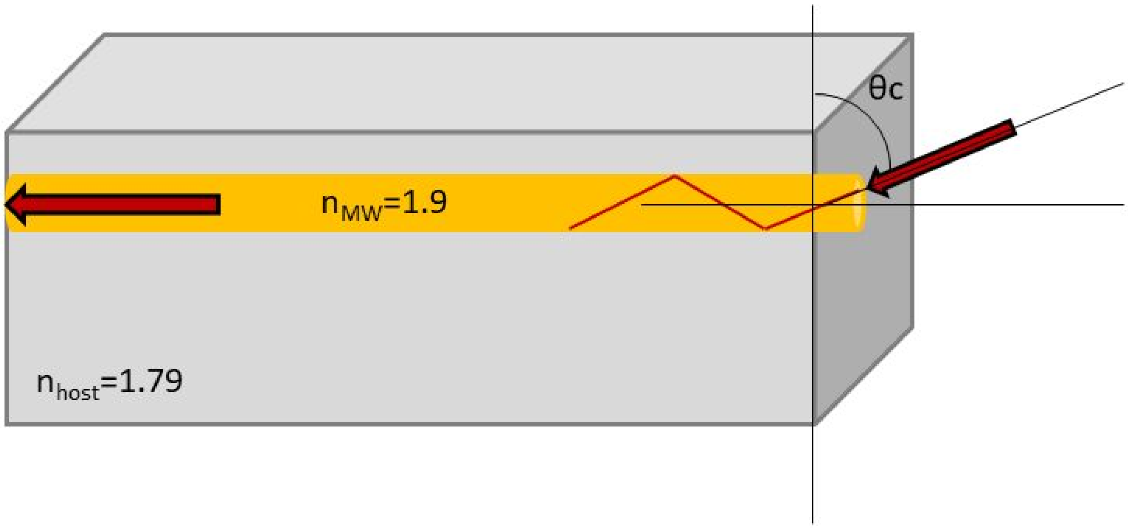

Fig. S3: Cross-section illustration of the waveguide device depicting the formation of the silver-rich channel upon incorporation of the 0.3AgI+$AgPO_3$ MW within the $AgPO_3$ host glass. The total internal reflection (TIR) principle is also depicted.

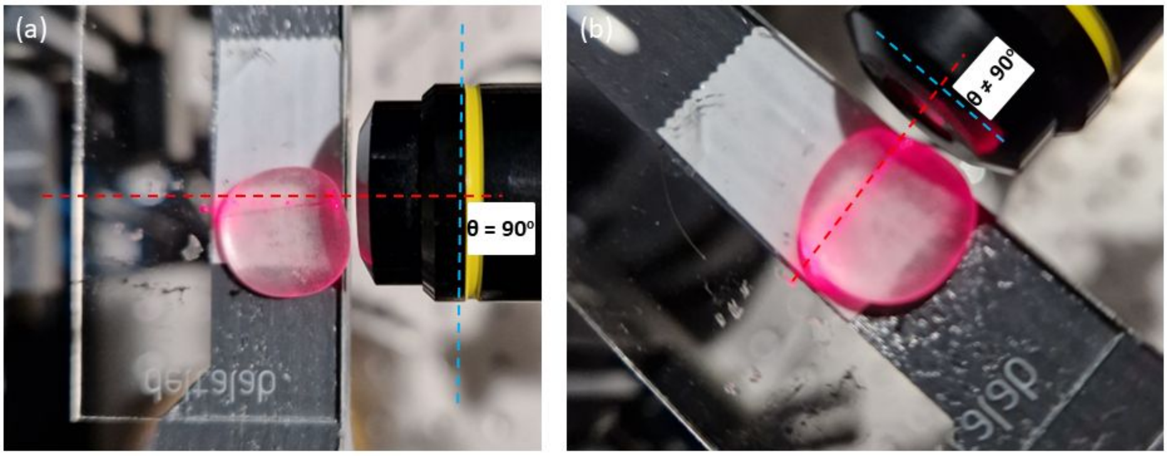

Fig. S4: (a) Red laser beam focused outside the waveguide pathway, and thus, forming a 90° angle with respect to the objective. **(b)** Red laser beam coupled-in the waveguide pathway, and thus, deviating from the vertical orientation with respect to the objective since the light propagates through the silver-rich waveguide pathway.

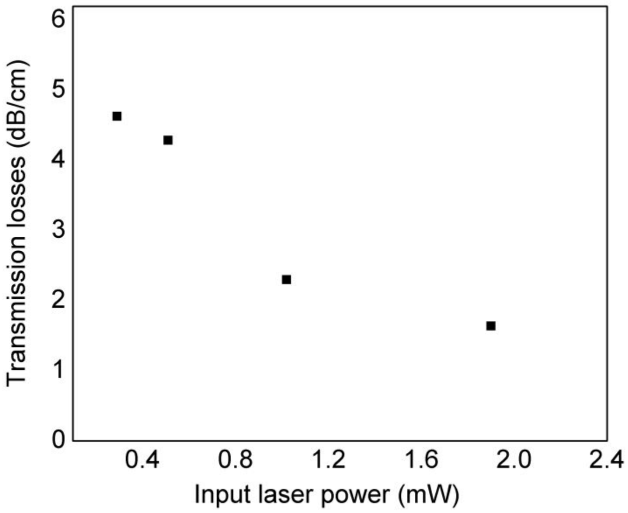

Fig. S5: Optical losses for various applied powers of the coupling-in light.

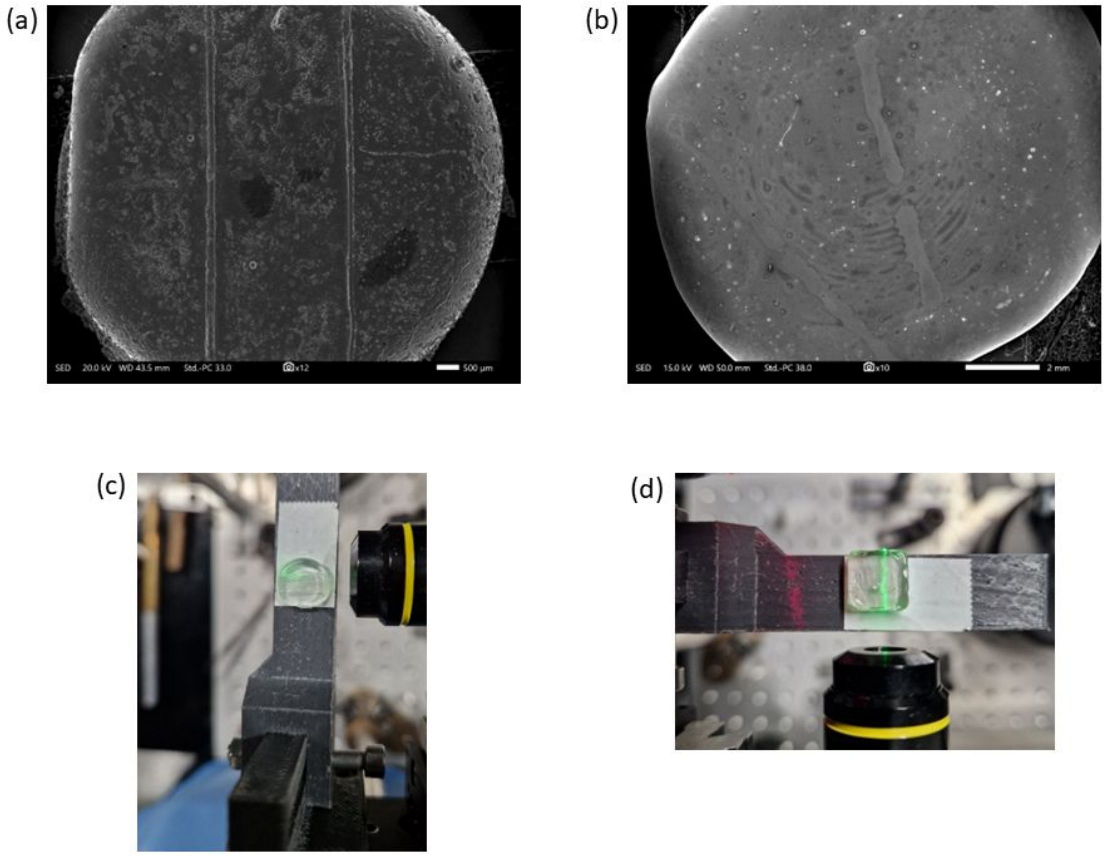

Fig. S6: (a) Scanning electron microscopy (SEM) image of the multipath waveguide with parallel MWs. **(b)** SEM image of the multipath waveguide device with one MW placed diagonally. **(c)** Waveguide device with only green light propagating through the one for the parallelly positioned MWs. **(d)** Waveguide device with only green light propagating through the parallel incorporated MW, whereas as the other is positioned diagonally.

Videos:

Vid. S1: Demonstration of the microwire (MW) drawing process from the splat-quenched $0.3AgI+0.7AgPO_3$ glass.

Vid. S2: Demonstration of the light coupling-in process to the single waveguide device, upon moving the green laser beam with the micrometric stage towards the input point.
